# Rationale and design of a randomised clinical trial for an extended cardiac rehabilitation programme using telemonitoring: the TeleCaRe study

**DOI:** 10.1186/s12872-016-0345-9

**Published:** 2016-09-06

**Authors:** Johan A. Snoek, Esther P. Meindersma, Leonie F. Prins, Arnoud W. J. van’t Hof, Maria T. Hopman, Menko-Jan de Boer, Ed P. de Kluiver

**Affiliations:** 1 Sports Medicine Department, Isala, Dokter Van Heesweg 2, 8025 AB Zwolle, The Netherlands; 2 Cardiology Department, Isala Heart Centre, Zwolle, The Netherlands; 3 Cardiology Department, Radboud UMC, Nijmegen, The Netherlands; 4 Diagram, Zwolle, The Netherlands; 5 Physiology Department, Radboud UMC, Nijmegen, The Netherlands

**Keywords:** Cardiac rehabilitation, Telemonitoring and telecoaching, VO2, Cardio respiratory fitness, Leisure time physical activity, Randomised controlled trial

## Abstract

**Background:**

Despite the known positive effects of cardiac rehabilitation and an active lifestyle, evidence is emerging that it is difficult to attain and sustain the minimum recommendations of leisure time physical activity. The long-term benefits are often disappointing due to lack of adherence to the changes in life style. Qualitative research on patients’ perspectives suggests that motivation for lifestyle change tends to diminish around 3 months after the index-event. The time most cardiac rehabilitation programmes end. The aim of the present study is to determine if prolongation of a traditional cardiac rehabilitation programme with additional heart rate based telemonitoring guidance for a period of 6 months results in better long term effects on physical and mental outcomes, care consumption and quality of life than traditional follow-up.

**Methods:**

In this single centre randomised controlled trial 120 patients with an absolute indication for cardiac rehabilitation will be randomised in a 1:1 ratio to an intervention group with 6 months of heart rate based telemonitoring guidance or a control group with traditional follow-up after cardiac rehabilitation. The primary endpoint will be VO2_peak_ after 12 months. Secondary endpoints are VO2_peak_ after 6 months, quality of life, physical-, emotional- and social functioning, cardiac structure, traditional risk profile, compliance to the use of the heart rate belt and smartphone, MACE and care-consumption.

**Discussion:**

The TeleCaRe study will provide insight into the added value of the prolongation of traditional cardiac rehabilitation with 6 months of heart rate based telemonitoring guidance.

**Trial registration:**

Dutch Trial Register: NTR4644 (registered 06/12/14).

## Background

Despite the known positive effects of Cardiac Rehabilitation (CR) and an active lifestyle, evidence is emerging that it is difficult to attain and sustain the minimum recommendations of Leisure Time Physical Activity (LTPA) [1, 2]. The long-term benefits of CR are often disappointing due to lack of adherence to the changes in life style [3, 4].

Cardiovascular diseases (CVD) are the leading cause of death and a major cause of disability and lost productivity in adults worldwide [5]. The aging and growth of the population have offset reduction in cardiovascular mortality in the past decade [6].

Both LTPA and Cardio Respiratory Fitness (CRF) are inversely correlated with mortality [7]. Lee et al. evaluated the long-term effects of changes in CRF on cardiovascular mortality. They observed a significant reduction in cardiovascular mortality of 27 % and 42 %, respectively (compared to those with a loss of CRF) in individuals who had either no change or improvements in CRF. The mean interval between the baseline and last examination being 6.3 years. For every 1-MET (Metabolic Equivalent of Task) increase in CRF over time, all-cause and cardiovascular mortality were reduced by 15 % and 19 %, respectively, showing that even small improvements in CRF can have a substantial effect [8].

Qualitative research on patients’ perspectives suggests that motivation for lifestyle change tends to wane around 3 months after the event, when the initial shock has worn off, and most patients start feeling better [9]. In addition to the already low uptake of CR in the Netherlands median total training duration seems to be less than 3 months [10, 11]. Consolidating lifestyle habits require continued attention and appropriate guidance [3]. The transition of a supervised and structured training programme to a continuation without this guidance could be a possible explanation for the low compliance [12]. A better self-efficacy by telemonitoring could help them to be engaged in and actually perform healthy behaviour (e.g. exercise adherence).

The main aim of the present study is to determine whether prolongation of a traditional CR programme with heart rate based telemonitoring guidance for a period of 6 months results in better long-term effects on physical and mental outcomes, care consumption and quality of life (QOL). In order to see whether patients are able to sustain their CRF and LTPA levels after the telemonitoring intervention of 6 months, this will be followed by a 6-month period without telemonitoring guidance.

The primary objective of the study is to assess whether the addition of 6 months of heart rate based telemonitoring guidance to the traditional CR programme results in a higher peak oxygen uptake (VO_2peak_) than a regular follow-up 12 months after CR. Secondary outcome parameters are: VO_2peak_ at 6 months, cardiac structure and function, QOL, physical (LTPA level), emotional and social functioning, traditional risk profile, compliance to the use of the smartphone, adherence to the minimum recommendations of LTPA, care consumption (hospital admissions, outpatient clinic visits, general practitioner (GP) visits, cardiovascular interventions and lab testing) and Major Adverse Cardiac Events (MACE).

## Methods

### Design

**Fig. 1:**
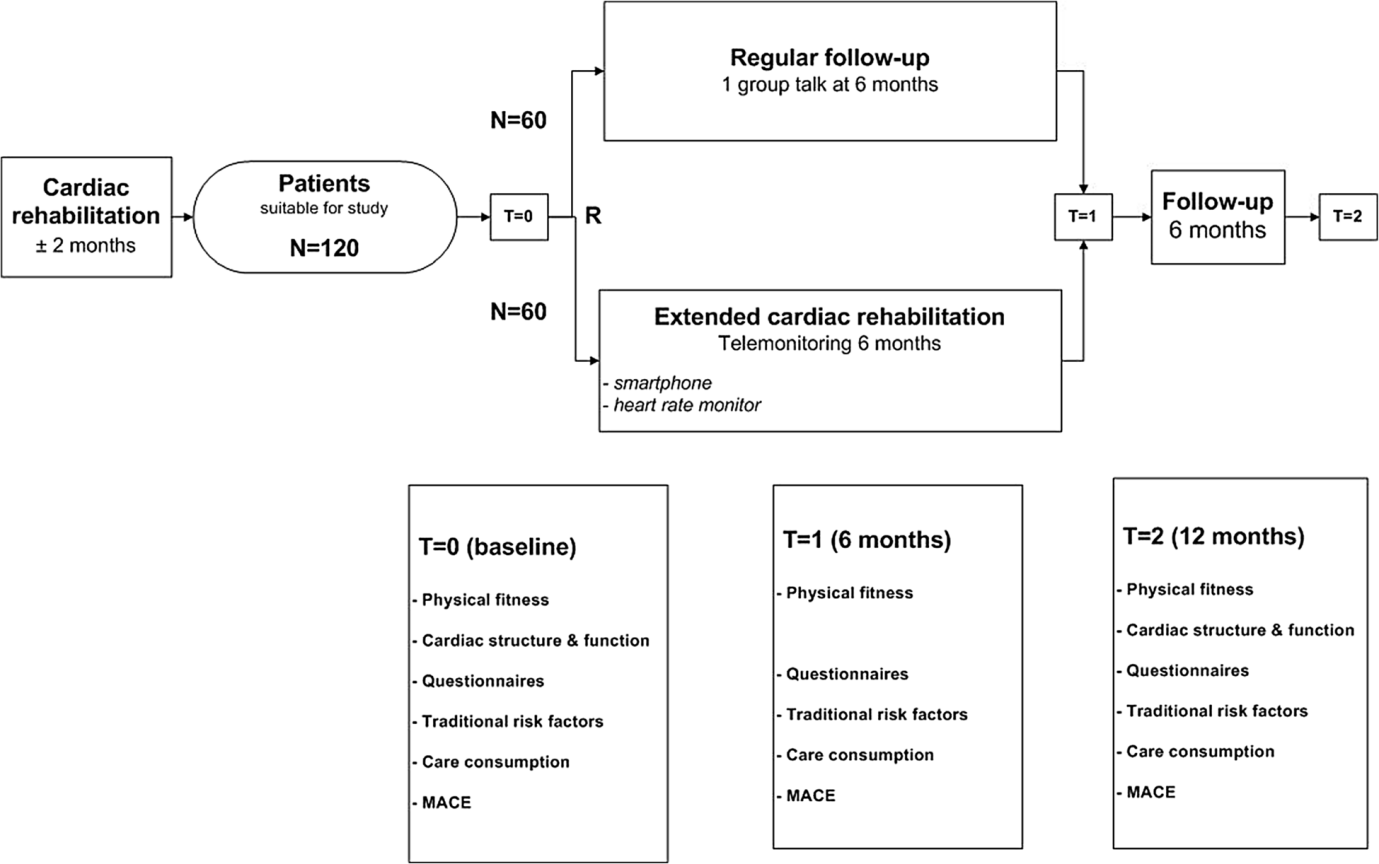
Flowchart, illustrating randomisation and testing at different time points

This study is designed as a prospective single centre randomised controlled trial. Patients will be evaluated at baseline (T0), after 26 weeks (T1) and 12 months (T2) after randomisation. Figure 1 provides a flowchart of the study protocol. The study complies with the World Medical Association Declaration of Helsinki on ethics in medical research and is approved by the medical ethics committee of Isala (NL48475.075.14; 14.0334). Clinical Trial Registration: NTR4644.

### Patients

The study will enrol in total 120 patients referred to the CR centre of Isala Zwolle. Patients with a minimal attendance of 80 % in the exercise programme, a signed written informed consent and one of the following indications for CR (acute coronary syndrome (ACS), percutaneous coronary intervention (PCI) or coronary artery bypass grafting (CABG) within 3 months prior to the start of the CR programme) will be included. All patients assessed for eligibility will be registered. Exclusion criteria are a contraindication to CR according to the Dutch national guideline, mental impairment leading to inability to cooperate, severe impaired (musculoskeletal) ability to exercise, signs of cardiac ischemia and/or positive exercise testing on cardiac ischemia, insufficient knowledge of the Dutch language, no access, availability or insufficient knowledge of a computer with internet or an implanted cardiac device (pacemaker, ICD) [13].

### Sample size

Preliminary calculations suggest that for an expected difference in VO_2peak_ of 3.0 ml/kg/min between the control and intervention group with an estimated standard deviation of 4.8 ml/min/kg, assuming 90 % power and 5 % significance level (α = 0.05), a total of 108 patients is needed to detect a beneficial effect of telemonitoring. These numbers are based on a previous study that shows the effects of 52 weeks of CR with combined supervised and unsupervised exercise sessions [14]. Taking into account a 10 % dropout we plan to randomise 60 patients in each group.

### Randomisation

At the end of the regular CR programme, patients meeting the in- and exclusion criteria will be informed and requested to participate. After receiving written informed consent, patients will be randomised in a 1:1 ratio to the intervention group with telemonitoring guidance or the control group with a traditional follow-up programme by means of a computerised allocation system applying an algorithm that prevented the care providers or the investigators from predicting the outcome of the randomisation process. This means that the person, who determines if the patient is eligible, at the time of this decision, is unaware of which group the patient will be allocated to. The patient and care providers are not blinded to the intervention.

### Telemonitoring/coaching

Patients in the intervention group are equipped with a smartphone (Samsung Galaxy Ace GT-s5830i, Korea) and Bluetooth connected heart rate monitor (Zephyr, Annapolis, USA). Therefore the device is able to register training mode, training time and also training intensity. The subjects receive a personal moderate to high intensity heart rate zone based on their Cardio Pulmonary Exercise Test (CPET) at T0 and are free to choose which type of exercise they are going to do. Moderate intensity will be defined as an intensity above their individual first ventilatory threshold [15]. The patients in the intervention group will be advised to exercise at least 30 min a day 5 days a week in the above mentioned training zone. During exercise, type of exercise, heart rate and duration will be registered. Afterwards, patients are requested to score their perceived exertion of training (BORG score). Data will be transferred to a secured website through a secured wireless connection (CE registration NL-CAOO2-2014-32838; ISO 27001). Patients will have access to their individual webpage and their training history on the smartphone. Here they are able to see how much and with which intensity they have exercised. They will be contacted by telephone weekly in the first month, every other week in the second month and from then on every month until T1. During these telephone calls (10 in total) patients receive further advice on how to improve their LTPA level. The nurses discuss the exercise data and use motivational interviewing to motivate and stimulate the patient.

### Traditional follow-up

Traditional follow-up after CR consists of one out-patient visit 3 months after hospitalisation. During this visit medication is checked, information about the diagnosis is given and possible complaints are discussed. If necessary addition testing and extra visits are scheduled.

### Cardio pulmonary exercise testing

Cardiopulmonary exercise testing will be performed every visit in both groups using individualised cycle ergometer ramp protocols on an electro-magnetically braked cycle ergometer (Lode Corrival, Lode, Groningen, the Netherlands). Twelve-lead ECG is recorded continuously and blood pressure is measured every 3 min. Oxygen consumption and CO2 production will be measured breath-by-breath by ventilatory gas exchange (Metalyzer 3b, Cortex Biophysics GmbH, Leipzig, Germany).

All patients start with 1 min of rest, thereafter 2 min of unloaded cycling, an individualised ramp protocol in order to reach VO2_peak_ between 8 and 12 min, 1 min of complete rest to measure Heart Rate Recovery (HRR) and finally a recovery period of 4 min. Patients will be encouraged to exercise until levelling off of VO2 despite an increase in workload and/or to a respiratory exchange ratio >1.10. Afterwards, patients are asked to quantify their rate of perceived exertion according to the original BORG score. Peak VO2 will be determined as the mean value of the final 30 s of exercise. The following criteria will be used to correlate the CPET at different time points: <100 ml increase VO2 in the last minute, RER (Respiratory Exchange Ratio) ≥ 1.10, BORG ≥ 17, HR (Heart Rate) > 85 % predicted.

### Medical history, physical examination and additional testing

Medical history, medication and co-morbidities will be screened every visit. During each visit physical examination length, weight and blood pressure (manual auscultation in seated position) will be measured. Waist and hip circumference will be measured and skinfold thickness will be determined (biceps, triceps, sub-scapular, supra-iliac) to calculate lean body mass [16]. Furthermore resting electrocardiogram (supine position) and lung function test will be executed [17].

### Echocardiography

Echocardiographic parameters for cardiac structure and function will be measured at T0 and T2.

#### Structure

From the 2-dimensional echocardiogram, diameter of the aorta, left atrium, left ventricle at end-diastole (LVED) and at end-systole (LVES), end-diastolic wall thickness of the intraventricular septum (IVS), and left ventricular posterior wall (LVPW) will be measured in the parasternal long-axis view, tracing of the area of the left atrium, and diameter of the vena cava inferior at end expiration will be measured in the subcostal view, respectively.

#### Systolic function

Ejection fraction will be calculated using the formula: (LVEDV–LVESV/LVEDV), where left ventricular end-diastolic volume (LVEDV) and left ventricular end-systolic volume (LVESV) from 2-dimensional echocardiograms in the apical 4-chamber view will be determined using the biplane Simpson rule method.

#### Diastolic function

Standard LV inflow pulsed-wave Doppler measurements at the mitral leaflet tips, including peak flow velocity of the early rapid filling wave (E[−wave]), peak flow velocity of the late filling wave due to atrial contraction (A[−wave]), the E/A ratio,(early deceleration time), and isovolumetric relaxation time will be measured to assess global diastolic function. In addition left atrium inflow pulsed wave velocity Doppler measurements will be obtained, including pulmonary venous flow velocities in both diastole (D) and systole (S), and the S/D ratio. To measure myocardial relaxation, pulsed-wave TDI measurements at the septal and lateral mitral annulus will be obtained.

### Questionnaires

Subjects will be asked to fill in several validated questionnaires to assess general health. This includes QOL (KVL-H), physical functioning (IPAQ-long version), emotional functioning (PHQ-9, HADS) and social functioning (MPSS) [18]. These questionnaires will be completed at baseline and after 6 and 12 months.

### Blood analysis

Venous blood samples will be obtained after an overnight fasting period at baseline, after 6 and 12 months. Total cholesterol, serum triglycerides, high-density lipoprotein, low-density lipoprotein, ApoB, HbA1c and glucose are analysed by the biochemical laboratory using standard procedures for the analyses.

### Adverse events

All adverse events will be registered and serious adverse events (SAE) will also be reported continuously to the Contract Research Organisation (CRO) Diagram. SAE are defined as cardiovascular mortality, all-cause mortality, near sudden cardiac death, ACS, CV intervention/surgery, CV hospital admission and CV emergency visit.

### Care consumption and MACE

The occurrence of events (cardiovascular mortality, all-cause mortality, near sudden cardiac death, ACS, CV Intervention/surgery, CV hospital admission, CV emergency visits) and consumption of care (admission days, outpatient clinic visits, GP visits, interventions, radiology, nuclear and lab testing) will be collected by a (monthly) telephone call with the participants throughout the study period and patients’ electronic medical files.

### Data handling

eDream®, an electronic data capture and clinical data management programme (electronic case report form) will be used to collect and store the data. The database will be hosted and secured on the servers of Diagram and is on-line accessible.

### Statistics

All parameters will be analysed on the intention-to-treat principle. All patients will be analysed in the group they are randomised to. Categorical variables will be summarised by frequency and percentages. Continuous variables will be summarised by mean and standard deviation as well as median and interquartile range.

The primary treatment comparison is telemonitoring group versus control group. Student’s t-tests will be used to examine whether the continuous variables are different between the intervention groups at baseline, at 6 months and at 12 months. When necessary the continuous variables will be transformed to obtain a normal distribution, or a non-parametric test will be used (Wilcoxon rank-sum test). Chi Square tests or Fisher’s exact tests as appropriate will be used to examine whether the categorical variables are different between the intervention groups at baseline, at 6 months and at 12 months.

Time until MACE will be described by Kaplan Meier curves. The Kaplan Meier curves will indicate the occurrence of MACE during 1 year of follow-up. Patients who do not experience an event or are lost to follow-up without an event are censored. Differences in survival distributions between the telemonitoring and control group will be tested using logrank tests.

A two-sided p-value of less than 0.05 will be considered to be statistically significant in all analyses. Analyses will be performed with Statistical Analysis System, SAS, version 9.3 and with the Statistical Package for the Social Sciences (SPSS, IBM) version 22.0.

## Preliminary results

Patient demographics and reasons to refrain from participation are shown in Tables 1 and 2. Preliminary results show that 122 of 299 (40.8 %) consecutive patients meeting the in- and exclusion criteria were willing to participate. They are on average 59 years of age and predominantly male (82,5 %). Main reasons to refrain from participation are the time investment and doubts concerning the added value.

**Table 1:** Patient demographics

	Screened	Included
	Number (%)	
Patients screened	299	122
Men	232 (77.7)	101 (82.8)
Women	67 (22.4)	21 (17.2)
Age (mean years)	63.7	59.6

**Table 2:** Reasons to refrain from participation

	Number (%)
Too time consuming	64 (36)
Not convinced of added value	40 (23)
Insufficient computer knowledge	25 (14)
Follow-up in a different location	12 (7)
Physical impairment	12 (7)
Too much traveling time	7 (4)
Problems with coping	6 (3)
Holiday > 1 month or abroad during follow up	5 (3)
Heart rate belt is too much stress	3 (2)
Unknown	3 (2)

## Discussion

The long term benefits of lifestyle interventions are often disappointing due to lack of adherence and persistent change in life style. Qualitative research on patients’ perspectives suggests that motivation for lifestyle changes tends to wane 3 months after the event. Most Dutch CR programmes though last less than 3 months.

Several studies showed that telehealth interventions after traditional CR provide an effective risk factor reduction in secondary prevention. They showed promising results in encouraging patients to stay active after the acute rehabilitation phase [19–21]. Moreover Frederix et al. and Kraal et al. showed that Telerehabilitation appears to be a feasible and effective additional and/or alternative form of rehabilitation, which is more effective and efficient than centre based CR alone [22, 23]. The TeleCaRe study differentiates itself from other telehealth studies by the length of the guidance and the follow up after the intervention in combination with the possibility to monitor type, volume and intensity of LTPA. Only a few studies looked at a follow-up period after the telemonitoring period without an intervention and as a result it is still unclear if telehealth interventions induce persistent lifestyle change. Moreover by measuring heart rate during exercise we are, contrary to studies using a motion sensor, able to monitor and coach patients to exercise in the right exercise intensity zone and correlate exercise intensity to outcome parameters [15, 24].

The TeleCaRe study evaluates the effects of extending a traditional CR programme with telemonitoring guidance for a period of 6 months with a follow-up of 6 months without support. The main aim of the study is to determine if this extension results in better long-term effects on physical and mental outcomes, care consumptions and QOL than the regular follow-up after cardiac rehabilitation.

## Abbreviations

ACS: Acute coronary syndrome
CABG: Coronary artery bypass grafting
CPET: Cardio pulmonary exercise test
CR: Cardiac rehabilitation
CRF: Cardio respiratory fitness
CRO: Contract research organisation
CVD: Cardiovascular diseases
GP: General practitioner
HADS: Hospital anxiety and depressions scale
HR: Heart rate
HRR: Heart rate recovery
ICD: Implantable cardioverter defibrillator
IPAQ: International physical activity questionnaire
IVS: Intra ventricular septum
LTPA: Leisure time physical activity
LVED: Left ventricle at end-diastole
LVES: Left ventricle at end-systole
LVPW: Left ventricular posterior wall
MACE: Major adverse cardiac events
MET: Metabolic equivalent of task
MPSS: Mood and physical symptom scale
PCI: Percutaneous coronary intervention
PHQ: Patient health questionnaire
QOL: Quality of life
RER: Respiratory exchange ratio
SAE: Serious adverse events
